# Positron emission tomography in the diagnosis of Whipple’s endocarditis: a case report

**DOI:** 10.1186/s13104-015-1022-2

**Published:** 2015-02-26

**Authors:** Sarah-Lyne Jos, Emmanouil Angelakis, Thierry Caus, Didier Raoult

**Affiliations:** URMITE CNRS-IRD 198 UMR 6236, Aix Marseille Université, Faculté de Médecine et de Pharmacie, 27 Bd Jean Moulin, 13385 Marseille, France; INSERM, ERI-12 (EA 4292), University of Picardie, Department of Cardiac Surgery, University Hospital Amiens, Avenue René Laënnec – Salouël, 80054 Amiens, France

**Keywords:** Positron emission tomography, *Tropheryma whipplei*, Culture-negative endocarditis

## Abstract

**Background:**

Whipple’s disease is a systemic infection that sometimes is associated with cardiac manifestations. The diagnosis of *Tropheryma whipplei* endocarditis is still the result of chance because there are no diagnostic criteria and clinical signs are often those of cardiac disease rather than infection.

**Case presentation:**

Culture-negative endocarditis was suspected in a non-febrile 77-year-old French woman from North France with a history of a graft replacement 4 years prior. Positron emission tomography revealed intense fluorodeoxyglucose uptake around the metal ring of the aortic graft. The valve was replaced, and *T. whipplei* was detected in a valve sample by molecular assays. Immunohistochemical staining of the valve for *T. whipplei* was also positive.

**Conclusion:**

The localization of infectious foci by positron emission tomography and systematically testing valve specimens for *T. whipplei* are promising for diagnosing Whipple’s disease.

## Background

Cardiovascular disease is the leading cause of death in the United States and worldwide [1]. Blood culture-negative endocarditis accounts for 2.5% to 31% of all cases of endocarditis [2]. Blood culture-negative endocarditis is a severe and difficult-to-diagnose disease, but our understanding of it has greatly improved over the past 2 decades [2]. *Tropheryma whipplei*, the causative agent of Whipple’s disease has been indicated as an agent of blood culture-negative endocarditis [3]. *T. whipplei* endocarditis differs from classic Whipple’s disease, which primarily affects the gastrointestinal system. The bacterium can also cause localized chronic infections such as spondylodiscitis, meningoencephalitis, uveitis, and pneumonia. *T. whipplei* endocarditis is an emerging clinical entity mostly observed in middle-aged and older men with arthralgia [3]. The diagnosis of *T. whipplei* endocarditis is based on molecular assays of surgically obtained heart valves [3]. Positron emission tomography (PET) is a promising tool for the identification of infectious foci, especially in culture-negative infected cardiovascular devices [4]. PET scanning has higher sensitivity than computed tomography (CT) for the evaluation of the extent and localization of infections [4]. We report one case of a culture-negative endocarditis localized by PET and diagnosed as *T. whipplei* endocarditis by molecular assays and histology.

## Case presentation

**Figure 1:**
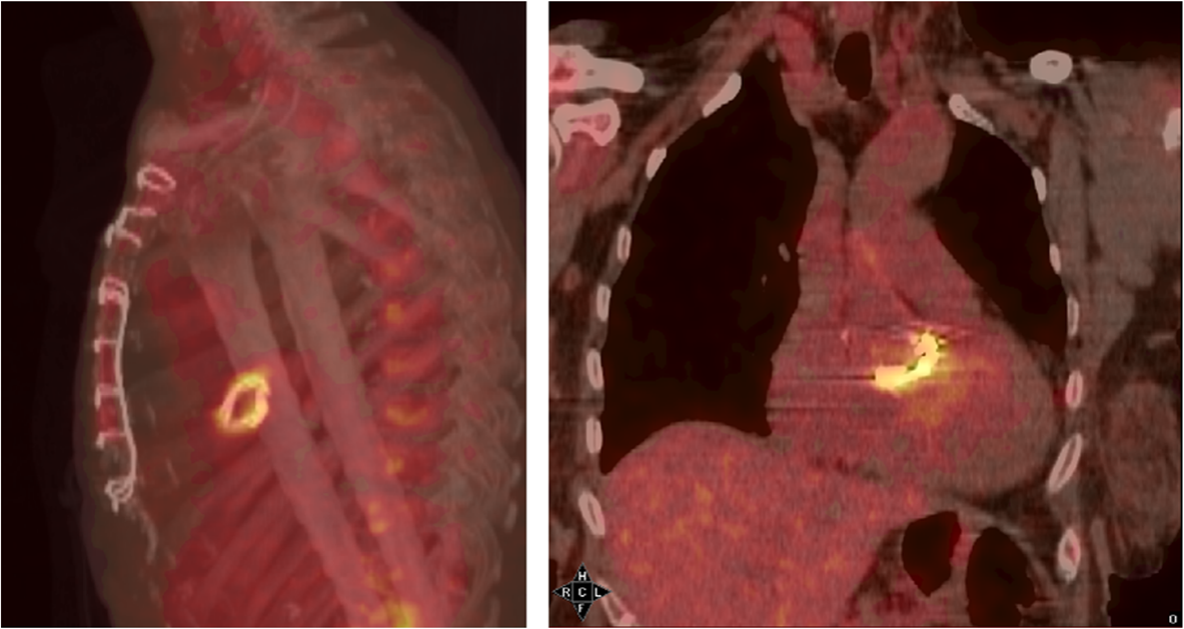
**Fluorodeoxyglucose uptake around the metal ring of the aortic graft revealed by PET scan.**

**Figure 2:**
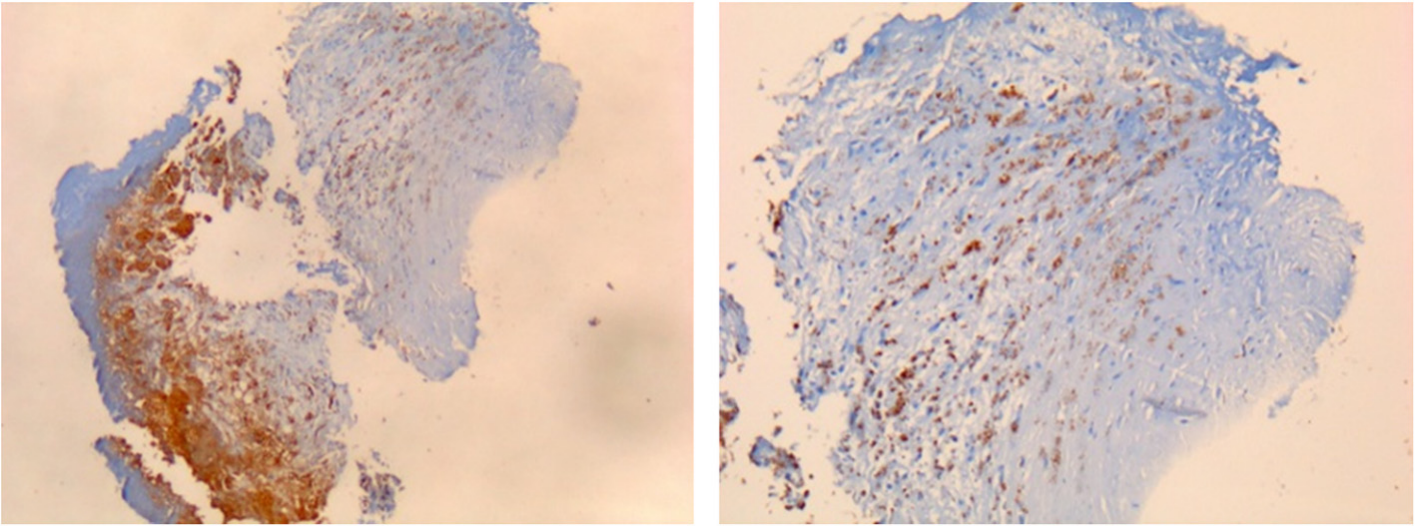
***T. whipplei*** **-positive immunohistochemical staining of the valve sample.**

A non-febrile 77-year-old French woman from North France was admitted to the hospital for faintness and neurological deficit with right hemiplegia and aphasia. The patient had a history of a graft replacement of her aortic valve 4 years before. Laboratory values revealed increased C-reactive protein and white blood cell count. Liver enzyme levels were normal. Brain magnetic resonance imaging (MRI) revealed two recent ischemic areas and CT scanning showed linear hypodensity in the posterior part of the spleen and two hypodense areas of the cortex of the right kidney. Based on the numerous ischemic zones, a cardiac cause was suspected. Transesophageal echocardiogram showed diffuse thickening of the bioprosthesis; mobile, high-potential-embolic, bioprosthesis stenosis; and intra-prosthetic aortic insufficiency. PET scan revealed intense fluorodeoxyglucose uptake around the metal ring of the aortic graft (Figure 1). The valve bioprosthesis was replaced, and treatment with amoxicillin and clavulanic acid (12 g/day) and gentamycin (120 mg 2 times per day) was started. Gram staining and standard cultures of the valve and blood were all negative. A valve sample was sent to our reference center in Marseille. DNA was extracted from this sample using a QIAamp tissue kit (Qiagen, Hilden, Germany) according to the manufacturer’s recommendations. Then, DNA extracts were tested by qPCR for the presence of *Tropheryma whipplei*, *Coxiella burnetti*, *Bartonella* sp., *Staphylococcus aureus*, *Enterococcus faecalis*, *Enterococcus faecium*, *Mycoplasma pneumonia*, *Streptococcus oralis*, *Streptococcus gallolyticus*, *Escherichia coli* and fungi [5]. All assays were negative except those for *T. whipplei*. A second qPCR assay targeting repeated sequences of *T. whipplei* [6,7] confirmed the diagnosis. In addition, PCR amplification and sequencing of the 16S rRNA gene [5] was also positive for *T. whipplei*. Genotyping was performed as previously described [8] and showed that this *T. whipplei* was genotype 16. Immunohistochemical staining of the valve for *T. whipplei* was positive (Figure 2) [9]. Culturing of the valve was negative. A course of 200 mg oral doxycycline once per day with 200 mg hydroxychloroquine three times per day for 18 months was introduced. The diagnosis for this patient was *T. whipplei* endocarditis in the context of a bioprosthetic aortic valve.

## Discussion

We report a case of *T. whipplei* endocarditis localized using PET scanning. The diagnosis of Whipple’s disease was then established by molecular assays and histology on the valve bioprosthesis. Infective endocarditis is associated with poor prognosis despite improvements in medical and surgical therapies [4]. Although the first description of *T. whipplei* endocarditis was made approximately 15 years ago, diagnosing this disease remains difficult because clinical signs are often those of cardiac disease rather than infection [3,10]. The first case was detected by chance when a broad-spectrum PCR was systematically applied to heart valve specimens [10]. PET scanning has been used to detect periprosthetic valve abscesses even in cases in which transesophageal echocardiography and transthoracic echocardiography were normal or doubtful, particularly in cases of prosthetic valve infections, in which results of initial echocardiography are not useful in 30% of cases [4]. The localization of infectious foci by PET had previously resulted in the diagnosis of only two cases of Whipple’s disease [11,12] and has been used to detect periprosthetic valve abscesses even in cases in which transesophageal echocardiography and transthoracic echocardiography were normal or doubtful [4]. In our case, PET scanning was especially valuable in the early diagnosis of *T. whipplei* endocarditis because it identified uptake at the graft replacement of the aortic valve, indicating an infection.

Blood culture-negative endocarditis accounts for 2.5%–31% of all cases of endocarditis. *T. whipplei* endocarditis is an emerging clinical entity mostly observed in middle-aged and older men with arthralgia [3]. Indeed, the disease occurs mainly in white men who are ~50 years of age with cardiac manifestations such as heart failure, acute ischemic stroke, and peripheral arterial embolism [3]. *T. whipplei* endocarditis is a frequent pathogen among cases of endocarditis, but its diagnosis is still the result of chance because there are no diagnostic criteria and clinical signs are often those of cardiac disease rather than infection [3,13]. In heart valves, *T. whipplei* is surrounded by an inflammatory process and inside the macrophages [3]. *T. whipplei*-infected heart valves show the typical histologic features of infective endocarditis: vegetations, inflammatory infiltrates, and valvular destruction. The diagnosis of *T. whipplei* endocarditis is based on molecular assays of surgically obtained heart valves [3]. The performance of repeat PCR for *T. whipplei* on blood specimens is a major criterion in the Duke classification for endocarditis.

Genotyping revealed that the valve was infected by *T. whipplei* genotype 16. *T. whipplei* genotyping has shown high genetic diversity unrelated to pathogenicity [14]. *T. whipplei* genotype 16 has been detected in the cerebrospinal fluid of a patient with neurological Whipple’s disease in Germany and in the synovial fluid of a patient with classic Whipple’s disease [8]. Moreover, it has been detected in the gastric juice of a patient without clinical manifestations from Switzerland [8]. In Europe, *T. whipplei* genotype 3 is the most common genotype and appears to be epidemic and specific to France, Switzerland, and Italy [8]. The second most common genotype in Europe is the genotype 1, which is endemic and mainly observed in central Europe.

## Conclusions

We report a case that illustrates the usefulness of ^18^FDG-PET/CT for diagnosis of *T. whipplei* infectious endocarditis in a patient with a graft replacement of the aortic valve. The localization of infectious foci by PET scanning and the systematic testing of valve specimens for *T. whipplei* are promising, and these procedures can be performed in patients of all ages by adjusting the dose of ^18^FDG to the weight of the patient. Thus, we believe that *T. whipplei* should be considered in the diagnosis of culture-negative endocarditis and that PET scanning might be helpful in the diagnosis of *T. whipplei* infectious endocarditis, although it will not replace clinical evaluation, laboratory tests, and echocardiography.

## Consent

Written informed consent was obtained from the patient for publication of this Case Report and any accompanying images. A copy of the written consent is available for review by the Editor-in-Chief of this journal.

## Abbreviations

PET: Positron emission tomography
MRI: Magnetic resonance imaging
CT: Computed tomography
